# Presence of immune factors in freshwater mussel (*Hyriopsis cumingii*) entails autologous serum an essential component in the culture of mantle cells

**DOI:** 10.3389/fimmu.2023.1173184

**Published:** 2023-05-05

**Authors:** Zhiyi Bai, He Wang, Xuenan Li, Xiaoya Shen, Yige Chen, Yuanshuai Fu, Wenjuan Li

**Affiliations:** ^1^ Key Laboratory of Freshwater Aquatic Genetic Resources, Ministry of Agriculture and Rural Affairs, Shanghai Ocean University, Shanghai, China; ^2^ Shanghai Engineering Research Center of Aquaculture, Shanghai Ocean University, Shanghai, China; ^3^ Shanghai Collaborative Innovation Center for Cultivating Elite Breeds and Green-culture of Aquaculture Animals, Shanghai, China

**Keywords:** *Hyriopsis cumingii*, cell culture, serum, proteomics, immune factors

## Abstract

Mussel cell culture is a challenging problem and serum serves a crucial biological role in cell culture as an autologous supply and an immunizing agent. In this study, the biology (calcium ions, total protein, pH, and osmotic pressure) of fetal bovine serum (FBS) and *Hyriopsis cumingii* serum (HCS) was investigated, and the development of *Hyriopsis cumingii* (*H. cumingii*) mantle cells in HCS and FBS systems was examined. The results showed that total protein, calcium ions, and osmotic pressure varied significantly (*p*<0.05). The activity of mantle cells was superior in the HCS culture system to that in the FBS culture system. The label-free technique was used to distinguish the two serum proteins to investigate the supportive effect of autologous serum on cell culture. These were examined for 109 unique proteins and 35 particular HCS proteins. Most differentially expressed proteins (DEPs) were involved in immune response, cell differentiation, and calcium ion binding. Furthermore, immune factors such as HSP, CALR, APOB, C3 were identified with significant differences. HSP was significantly more present in HCS than in FBS as an endogenous protective protein that regulates immune system function, cell differentiation, transport, and activity regulation. Parallel reaction monitoring (PRM) analysis was carried out to validate the expression levels of 19 DEPs, indicating high reliability of the proteomic results. This study reveals the important role of immune factors in mussel cell culture, providing a theoretical basis for explaining the applicability of autologous serum in cell culture. It is also helpful in improving the cell culture conditions of mussels.

## 1 Introduction

At least thousands of cell lines have been established so far. However, no successful cell line has been established in marine invertebrates (1). The addition of fetal bovine serum (FBS) to the culture medium of *Hyriopsis cumingii* mantle cells could keep the cells alive for about 10 days *in vitro* (2). Although the research method has been established, the long-term culture has not yet been realized, and the composition of the medium is still being explored. Clam lymphoid blood and FBS were added to the basal medium to prolong the cell survival time (3). Chicken serum was used for the first time in the mantle cell culture of *Mytilus galloprovincialis*, and the growth-promoting activity of chicken serum and FBS was compared. It showed that 20% chicken serum had the same effect as 30% FBS, which could maximize mitosis (4). The only established freshwater snail (*Biomphalaria glabrata embryonie)* (Bge) cell line in aquatic invertebrates was sensitive to serum components and required several attempts (5).

Serum is a complex mixture obtained by removing fibrin from plasma. It is a common addition to *in vitro* cell culture solution because it can provide adherent factors, immunoglobulin, insulin, growth and immune factors required for cell growth (6). FBS contains a variety of growth factors and cytokines that maintain cell growth and proliferation and is often used as a key component of cell culture (7). The best survival rate of *Chlamys farreri* mantle cells was obtained by using 20% FBS combined with inorganic salt (8). Adding FBS and insulin into *Mytilus edulis* larvae cells can maintain its viability for more than 2 months *in vitro* (9). Therefore, serum plays an important role in the composition of the medium.

Cell differentiation, proliferation, metabolism, senescence, and immune and apoptosis are physiological processes closely related to immune and growth factors (10). Proteins in the serum play a role in protecting and promoting the cells, an important component of humoral immunity. It contains a variety of important immune factors of mussel humoral immunity (the alpha-2 Macroglobulin (α2M) (11), lectin (12), heat shock proteins (HSPs) (13), and growth factors (Insulin-like growth factor/IGF (14), and basic fibroblast growth factor/bFGF), which are similar to the environmental conditions *in vivo*. Appropriate serum added to the culture medium promotes cell growth and maintains cell survival time *in vitro* (15).

The immune response greatly influences the quality of the pearl (16). Mussel immune system only has humoral immunity,playing a role in damage repair and antioxidants. Mussels have an open circulatory system. Therefore, serum is highly important for humoral immunity (12). There is a gap between FBS and autologous serum, and it is not clear how to reduce the excessive immune response in cell culture and improve cell survival time. Autologous serum can satisfy various physical and chemical factors of cell growth and survival (3, 17). Therefore, the selection of serum in invertebrate cell culture needs further study.

*H. cumingii* is an important economic freshwater mussel species with the largest yield in the world (18), producing more than 80% of the world’s freshwater pearl production (19). The mantle of the mussel is the protective organ of shellfish. It has the function of biological mineralization, which is important for pearl formation (20). However, no mature cell line of mantle cells limits the study of gene function verification, immunity, disease control, and pearl formation mechanism of shellfish. As an aquatic mollusk, *H. cumingii* has only innate immunity, among which humoral immunity is an important component (21). Serum is an important component of humoral immunity, in which immune factors and cytokines play an important role in fine culture. In this study, *H. cumingii* mantle cells were cultured separately in the medium supplemented with *H. cumingii* serum (HCS) and FBS to explore the appropriate environment. Label-free proteomics method was used to screen the differential proteins (DEPs) of immunity, growth and mineralization between HCS and FBS for functional analysis and PRM verified the results. Thus, the study aimed to explore biological characteristics of proteins between autologous serum and FBS, fill the gap of immune factors in HCS, and provide a new idea for optimizing the cell culture system of mussel.

## 2 Materials and methods

### 2.1 Experimental mussels and sample collection

The *H. cumingii* used in this study were cultured animals. All experiments on freshwater pearl mussels were conducted according to institutional and national guidelines. Healthy mussels (*H. cumingii*, 28 individuals) were obtained from a farm in Wuyi (Zhejiang, China) in March.

**Figure 1:**
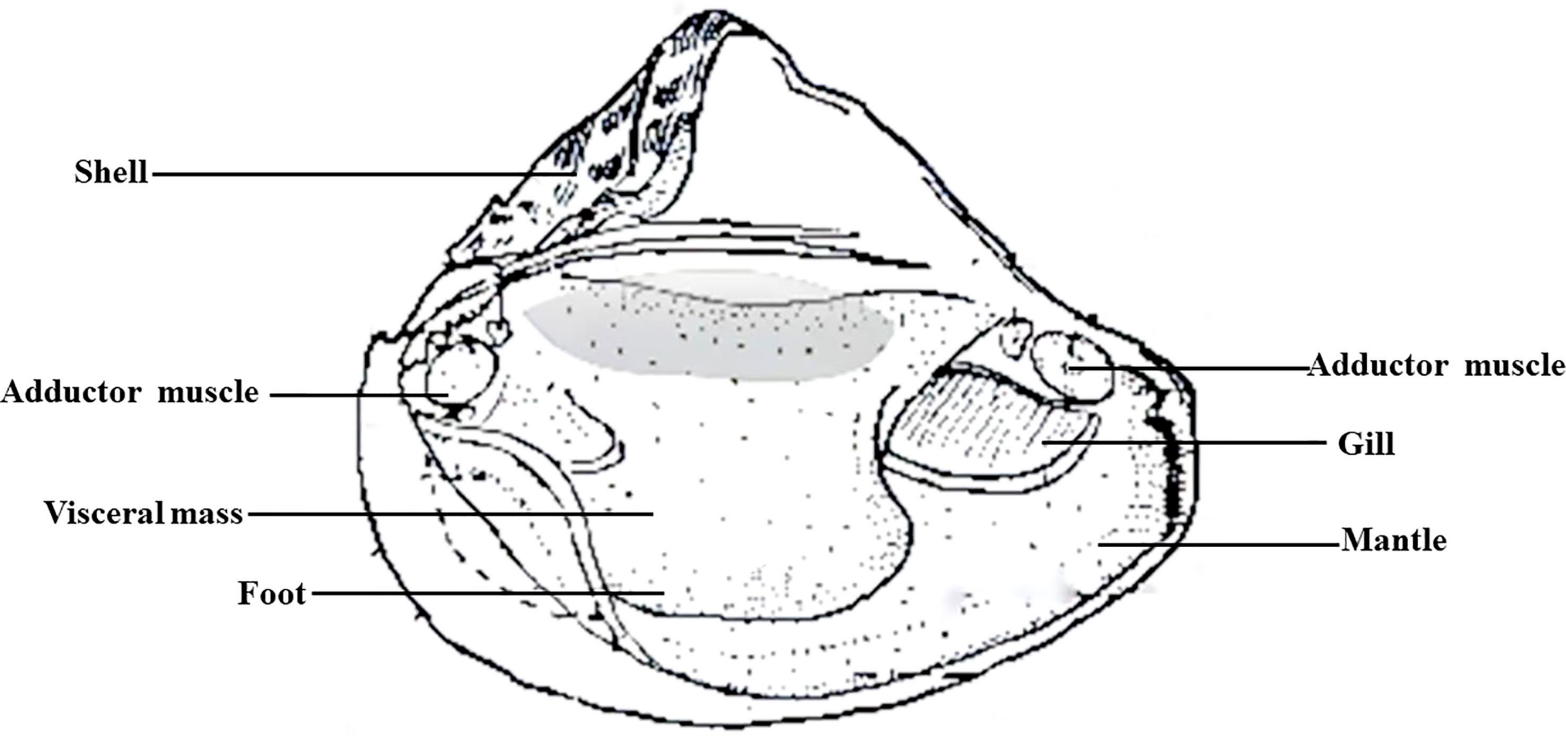
Tissue anatomical structure of *H. cumingii*.

Preparation of autologous serum of *H. cumingii*: 18 healthy mussels (6 mussels from 1-year-old laboratory-raised, 6 mussels from 1-year-old river-raised, 6 mussels from 3-year-old laboratory-raised) were sprayed with alcohol (75%) and left to stand for 15-20 min. Then, non-anticoagulated blood was drawn from the adductor site using a sterile 1 mL syringe (**Figure 1**). One mL of each mussel was collected, and 6 mL of serum from each group of 6 mussels was mixed into a 15 mL centrifuge tube and mixed 10 times by inverting up and down gently. The sample was immediately placed on ice for 10-20 min, centrifuged at 1500 g for 5 min to obtain the upper serum layer, and centrifuged at 1800 g for 5 min. The supernatant was transferred to a new tube and filtered through 0.45 μm and 0.22 μm membranes in a 1.5 mL centrifuge tube, centrifuged at 13,000 g for 2 min, and filtered through a 0.1 μm sieve. Samples were taken within 1 h, sealed, and stored at -20°C.

Preparation of cell sample suspensions: The mantle tissue was removed from the experimental mussel with a sterilized scalpel and washed with 10% phosphate-buffered solution (PBS) (Gibco, Waltham, MA, USA) 3-5 times successively and 75% alcohol solution for 10 s. Then, it was washed with three-resistance of 2%, 10%, 20%, 10%, and 2% successively, and each gradient for 10 min. Finally, the cleaned mantle tissue was placed in a 10% PBS buffer. The mantle tissue was then separated and cut into small pieces (1 mm^2^). After digestion in 0.25% trypsin at 37°C 12 h, excess tissue mass was filtered through 200 and 400 mesh strainers. Cell precipitates were collected after centrifugation at 800 rpm for 5 min. Two mL of 1640 medium was used to blow and precipitate evenly. Then, using a hemocytometer, the cells were counted. The cell number was adjusted to 5×10^5^−1×10^6^ mL^-1^ (diluted in 1640 culture medium). Cells were cultured at 25°C.

### 2.2 Cultivation of mantle primary cells of *H. cumingii* in different serum type groups

This experiment was designed with three different serum groups for the culture of mantle cells. Six replicates of 6-well plate petri dishes (127×85.5×23mm, Corning, the USA) were made for cell culture in each experimental group.

10% FBS group: RPMI-1640 was used as the base medium with 10% FBS, 2% triple antibody, 1% sodium pyruvate, 1% MEM non-essential amino acids, 15 ng/mL of basic fibroblast growth factor (bFGF), 2 ng/mL of epidermal growth factor (EGF), 1 ng/mL of insulin-like growth factors-1(IGF-1), and 0.1% 2-mercaptoethanol, and NaOH solution was used to adjust the pH to between 7.2 and 7.4. All reagents were purchased from Gibco, Waltham, MA, USA.

5% FBS & 5% *H. cumingii* autologous serum (HCS) group: 10% FBS was replaced with 5% FBS and 5% autologous serum in the 10% FBS group.

10% HCS group: 10% FBS was replaced with 10% *H. cumingii* autologous serum in the 10% FBS group.

The cell suspension was divided into three portions, and each portion was transferred to a culture dish (60×15mm). Five mL of the corresponding complete culture medium was added and incubated at 26°C. The above operations were carried out in the cell room to ensure a sterile environment. The growth of mantle cells was observed under an inverted microscope (Olympus, BH2, Tokyo, Japan) at 0 d, 1 d, 2 d, 3 d, 4 d, and 5 d in different serum groups and photographed and recorded. According to the instructions of the cell counting KIT-8 kit (C0038, Beyotime, Shanghai, China) (22), the viability of *H. cumingii* mantle cells was detected, with 6 replicates in each group. The control hole value was determined using the M199 medium (23).

### 2.3 Measurement of autologous serum of *H. cumingii* physiological indicators

Total serum protein values were determined using the BCA method (24), and the method was determined by referring to the instructions of the kit developed by Shanghai Biyuntian, Shanghai, China. Ca^2+^ concentration was determined by the methyl thymol blue (MTB) method (25). The determination method was in accordance with the kit instruction developed by the Nanjing Jiancheng Institute of Biological Engineering, Nanjing, China. The osmolality value was determined using a fully automatic freezing point osmometer (Shanghai Medical University Instruments, FM-8P, Shanghai, China). pH measurement was used a digital pH meter (Lithium Cabinet, PHS-25).

### 2.4 Protein extraction, quantification, and identification

Eighteen mussels with an average age of 2 years from different locations were used as the experimental group, and purchased FBS was used as the control group. The HCS was obtained from Wuyi (Zhejiang, China), and the method of serum collection was referenced in 2.1. FBS was purchased from Gibico (Waltham, MA, USA). There were three replicates for each serum group. SDT buffer (4%SDS,100 mM Tris-HCl, pH 7.6) was added to the sample and transferred to 2 mL tubes with an amount of quartz sand. The lysate was homogenized by MP Fastprep-24 Automated Homogenizer (6.0M/S, 30s, twice). The homogenate was sonicated and then boiled for 15 min. After centrifuging at 14000 g for 40 min, the supernatant was filtered with 0.22 µm filters. The filtrate was quantified with the BCA Protein Assay Kit (P0012, Beyotime, Shanghai, China). The sample was stored at -20°C (26).

20 µg of proteins for each sample were mixed with 6X loading buffer respectively and boiled for 5min. The proteins were separated on 12% SDS-PAGE gel. Protein bands were visualized by Coomassie Blue R-250 staining.

### 2.5 Filter-aided sample preparation

Proteins (200 μg) for each sample were reduced with 50 mM DTT for 30 min at 56°C. Then, detergent, DTT, and other low-molecular-weight components were removed using UA buffer (8MUrea, 150 mM Tris-HCl pH 8.5) by repeated ultrafiltration (Sartorius, USA, 30 kD). Then, 100 μL iodoacetamide (100 mM IAA in UA buffer) was added to block reduced cysteine residues, and the samples were incubated for 30 min in darkness. The filters were washed with 100 μL UA buffer three times and then with 100 μL 25 mM NH4HCO3 buffer twice. Finally, the protein suspensions were digested with 4 μg trypsin (Promega, Madison, WI, USA) in 40 μL 25 mM NH4HCO3 (Baomanbio, China) buffer overnight at 37°C, and the resulting peptides were collected as a filtrate (27).

### 2.6 Liquid chromatography–mass spectrometry analysis

LC-MS/MS analysis was performed on a Q Exactive mass spectrometer (Thermo Fisher Scientific, Waltham, MA, USA) that was coupled to Easy nLC (Thermo Fisher Scientific, Waltham, MA, USA) (28). Peptide (2 μg) was loaded onto the C18-reversed phase analytical column (Thermo Fisher Scientific, Acclaim PepMap RSLC 50umX 15cm, nano viper, P/N164943, Waltham, MA, USA) in buffer A (0.1% formic acid) and separated with a linear gradient of buffer B (80% acetonitrile and 0.1% formic acid) at a flow rate of 300 nL/min. The linear gradient was as follows: 5% B for 5 min, 5-28% buffer B for 90 min, 28-38% buffer B for 15 min, 38-100% buffer B for 5 min, and hold in 100% buffer B for 5 min. MS data were acquired using a data-dependent top10 method, choosing the most abundant precursor ions from the survey scan (350–1800 m/z) for HCD fragmentation. MS1 scans were acquired at a resolution of 70,000 at m/z 200 with an AGC target of 3e6 and a maxIT of 50 ms. MS2 scans were acquired at a resolution of 17,500 at m/z 200 with an AGC target of 2e5 and a maxIT of 45 ms, and the isolation width was 2 m/z. Only ions with a charge state between 2-6 and a minimum intensity of 2e3 were selected for fragmentation. Dynamic exclusion for selected ions was 30 s. The normalized collision energy was 27eV.

The MS data were analyzed using MaxQuant software version 1.6.14.0. The RAW file was submitted to the MaxQuant server when searched, an established database, UniProt Felinae.fasta was selected, and the database search was performed (29). False discovery rate (FDR) of protein and peptide ≤ 0.01. DEPs in the FBS and HCS groups were screened according to difference multiples >= 2 or <= 0.5, *p*-value<0.05. Proteome Discoverer (Thermo Electron, version 2.2) was used for protein identification. The transcriptome database of *H.cumingii* mantle (30, 31) and FBS (https://www.ncbi.nlm.nih.gov) was used as a reference for the analysis. In protein quantification, the proteins must contain unique peptides. Only peptides with more than 95% reliability and proteins containing at least one unique peptide were used for protein identification. According to the multiple differences in data analysis, the data with at least two non-null values in the three repeated experiments were statistically tested and screened for DEP. DEPs were defined by differential ploidy (ploidy≥2or ≤ 0.5, *P*-values ≤ 0.05) for upregulation and downregulation of expression, respectively (29).

### 2.7 Bioinformatics analysis

Gene Ontology (GO) annotation proteome was derived from the UniProt-GOA database (http://www.ebi.ac.uk/GOA/) (28). All protein sequences were aligned to the *Homo sapiens* (see project report) database downloaded from the National Center for Biotechnology Information (NCBI) (ncbi-blast-2.2.28+-win32.exe), and only the sequences in the top 10 and E-value ≤ 1e-3 were kept. Secondly, the GO term of the sequence was selected with the top Bit-Score by Blast2GO. Then, the annotation from GO terms to proteins was completed by Blast2GO Command Line. Based on GO annotation, the proteins were classified into three categories: biological process, cellular component, and molecular function. Fisher’s exact test was used to enrich GO terms by comparing the number of DEPs and total proteins correlated to GO terms. GO categories with corrected *P*-values<0.05 were considered statistically significant.

Pathway analysis was performed using the Kyoto Encyclopedia of Genes and Genomes (KEGG) database. Fisher’s exact test was used to identify the significantly enriched pathways by comparing the number of DEPs and total proteins correlated to pathways (32). The pathway with a corrected *P*-value<0.05 was considered significant.

Subcellular localization of differential proteins was performed using the software WoLF PSORT. The protein-protein interaction network was analyzed using STRING (https://string-db.org/) (28).

### 2.8 Quantification of targeted proteins with parallel reaction monitoring

To verify the protein expression levels obtained label-free, 20 proteins were selected for PRM validation. These proteins were significantly more significant in the two serums and annotated in multiple pathways, such as immunity, growth, and mineralization. These proteins were extracted and enzymolized by label-free analysis. The tryptic peptides were dissolved in 0.1% formic acid (solvent A) and loaded onto a homemade reversed-phase analytical column. The gradient was comprised of an increase from 6% to 23% solvent B (0.1% formic acid in 98% acetonitrile) over 38 min, 23% to 35% in 14 min, climbing to 80% in 4 min, and then holding at 80% for the last 4 min at a constant flow rate of 700 nL/min on an EASY-nLC 1000 UPLC system. The peptides were subjected to an NSI source followed by tandem mass spectrometry (MS/MS) in Q ExactiveTM Plus (ThermoFisher Scientific, Waltham, MA, USA) coupled online to the UPLC (33).

### 2.9 Statistical analysis

All statistical analyses were performed using the SigmaPlot 10.0. statistical analysis software. Two tailed Student’s t-test was used for comparisons between two groups, and one-way analysis of variance (ANOVA) followed by Tukey–Kramer *post hoc* test was used for comparisons among three or more groups. For assessment between two independent variables, two way ANOVA followed by Tukey’s *post hoc* test was used. P values<0.05 were considered statistically significant.

## 3 Results

### 3.1 Microscopic observations on the *in vitro* culture of mantle cells of *H. cumingii* in different serum groups

**Figure 2:**
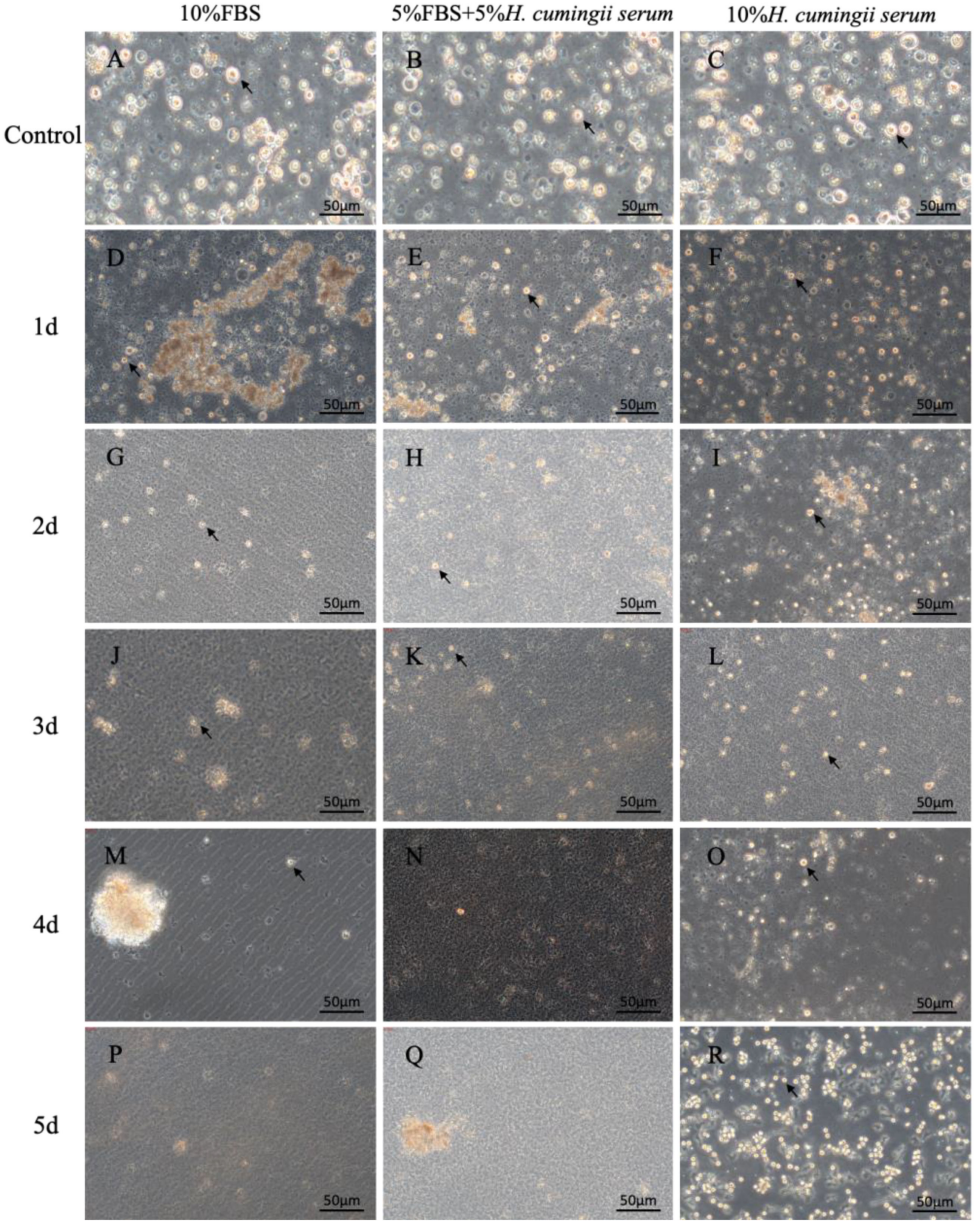
The microscopic observation of mantle cells. **(A)** Microscopic observation of cells cultured in 10%FBS for 0 d; **(B)** Microscopic observation of cells cultured in 5%FBS&5% *H.cumingii* serum for 0 d; **(C)** Microscopic observation of cells cultured in 10% *H.cumingii* serum for 0 d; **(D)** Microscopic observation of cells cultured in 10%FBS for 1 d; **(E)** Microscopic observation of cells cultured in 5%FBS&5% *H.cumingii* serum for 1 d; **(F)** Microscopic observation of cells cultured in 10%H.cumingii serum for 1d; **(G)** Microscopic observation of cells cultured in 10%FBS for 2d; **(H)** Microscopic observation of cells cultured in 5%FBS&5% *H*. *cumingii* serum for 2 d; **(I)** Microscopic observation of cells cultured in 10% *H*. *cumingii* serum for 2 d; **(J)** Microscopic observation of cells cultured in 10%FBS for 3 d; **(K)** Microscopic observation of cells cultured in 5%FBS&5% *H.cumingii* serum for 3 d; **(L)** Microscopic observation of cells cultured in 10% *H.cumingii* serum for 3 d; **(M)** Microscopic observation of cells cultured in 10%FBS for 4 d; **(N)** Microscopic observation of cells cultured in 5%FBS&5%*H. cumingii* serum for 4 d; **(O)** Microscopic observation of cells cultured in 10% *H*. *cumingii* serum for 4 d; **(P)** Microscopic observation of cells cultured in 10%FBS for 5 d; **(Q)** Microscopic observation of cells cultured in 5%FBS&5% *H.cumingii* serum for 5 d; **(R)** Microscopic observation of cells cultured in 10%*H.cumingii* serum for 5 d. Scale: 50 μm; The arrow points to the cell.

After microscopic observation, the number of cultured cells in the three different serum groups decreased at the beginning, and the growth rate of cells in the HCS group gradually accelerated in the late stage, showing a trend of concentrated growth and cell mass formation, while the other two groups gradually apoptotic. (**Figure 2**). In the 10% FBS group, after 1 d of cell culture, the cells were already reduced, and the morphology of the mantle cells was observed under an inverted microscope as round shape with a yellow granular substance in the center (**Figures 2A–D**). After 2 d of cell culture, the state of the cells began to deteriorate gradually. The cells were disintegrated, and no intact cell form could be seen (**Figures 2A–G, J**). After 4d of culture, it was obvious that the fragments of broken cells were glued together (**Figures 2A–M, P**). In the 5% FBS & 5% HCS group, there was a similarly large reduction in the number of cells on 1 d in culture, with only a few sporadically visible cells, and their survival quality was low. (**Figures 2A–E**). On 2 d in culture, the cells died and were gradually disintegrated, with no visible cell morphology (**Figures 2A–H**). After 3 d of cell culture, the disintegrated cells aggregated into flocs and floated in the upper layers of the medium (**Figures 2A–K**). In the 10% HCS group, there was also a large decrease in cells on 1 d of culture, but after 2 d of culture, a small number of irregularly shaped cells began to appear (**Figures 2A–C, F, I**). After 3 d of cell culture, the irregular cells increased in large numbers (**Figures 2A–L**). After 4 d of cell culture, the cell morphology began to turn into a round or oval shape, the yellow granular substance in the center was clearly visible, and the cell density increased significantly. The cell density increased significantly. At the same time, the cells began to grow against the wall, showing a good growth state, growing in clusters. (**Figures 2A–O, R**).

### 3.2 Activity analysis of *H. cumingii* mantle cells in different serum groups

**Figure 3:**
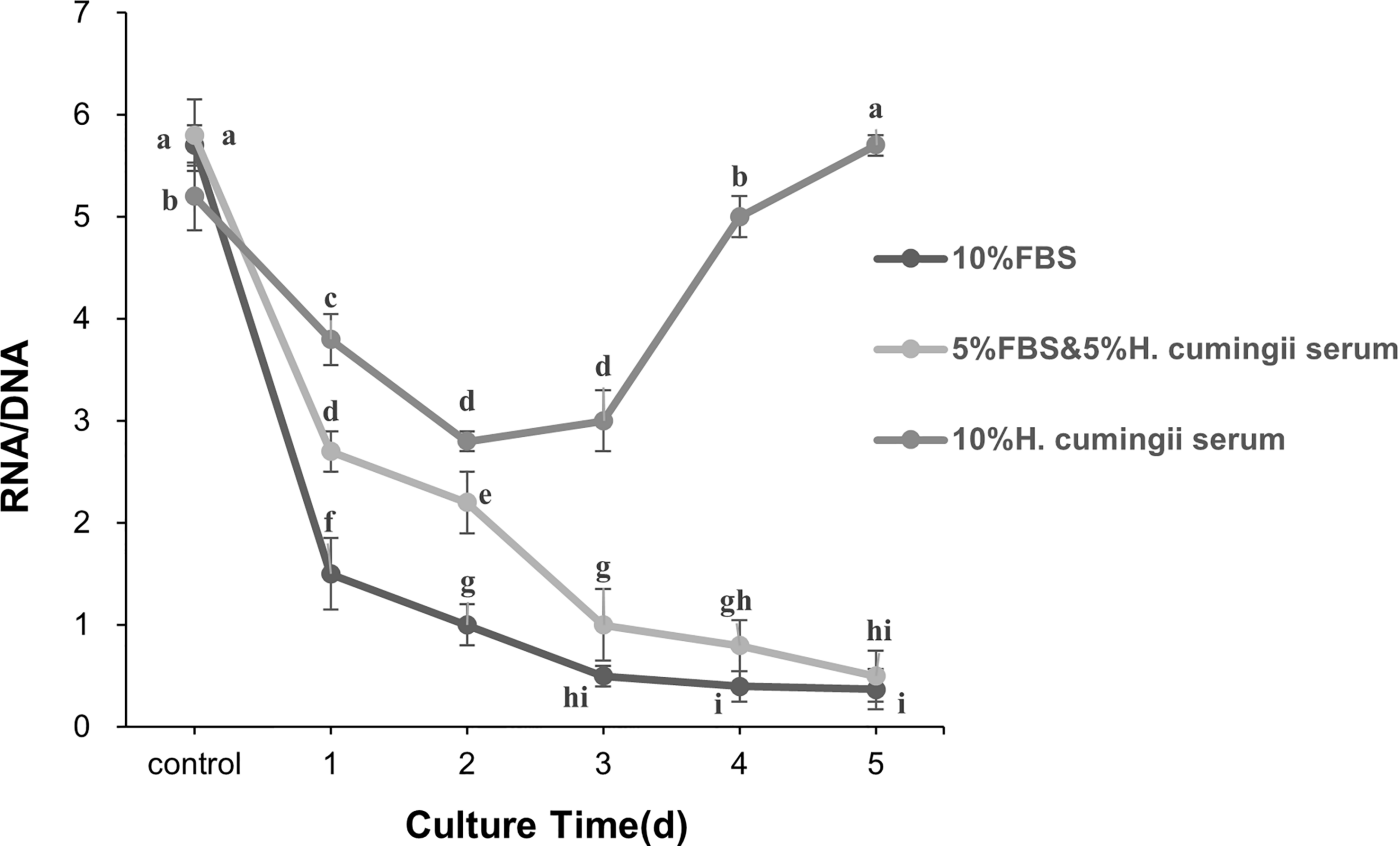
Changes in RNA/DNA ratio during the culture of *H. cumingii* mantle cells. Different letters represent significant differences between RNA/DNA values (*P*<0.05).

The RNA/DNA values and the mantle cell activity of the *H. cumingii* were significantly changed in three different serum groups during cell culture time (N > 6). The RNA/DNA ratio of cells in the three different serum groups decreased significantly from 0 to 2 d of culture (*P*<0.05), and the RNA/DNA ratio of cells in the 10% FBS and 5% FBS & 5% HCS groups showed the same trend, with a continuous decrease in cell viability. After 2 d of culture, the RNA/DNA ratio rose, and cell viability increased in the 10% HCS group. After 3 d of culture, the RNA/DNA ratio rose sharply, and cell viability increased significantly (*P*<0.05), indicating a continuous upward trend in cell viability. The HCS group had significantly higher cell activity than the other two groups (*P*<0.05, 11 times higher than the other two groups, **Figure 3**).

### 3.3 Serum physicochemical analysis

**Figure 4:**
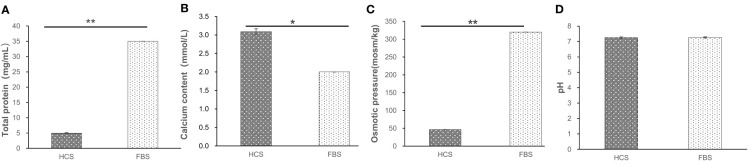
Various biological indicators of different serums. **(A)** Total protein of serum **(B)** Calcium content of serum **(C)** Osmotic pressure of serum **(D)** pH value of serum. *means difference (*P*<0.05), **mean significant difference (*P*<0.01).

The present study showed significant differences between the HCS and FBS groups in calcium (*P*<0.05). Total proteins concentration and osmotic pressure to HCS were significantly lower compared with FBS (*P*<0.01). There was no significant difference in pH between the two groups. The total protein was 7 times lower than FBS, and osmotic pressure was about 6 times lower. However, the calcium content of HCS was significantly different from that of FBS (*P*<0.05), which is 1.5 times higher than that of FBS (**Figure 4**).

### 3.4 Protein quality control and differential protein screening

**Figure 5:**
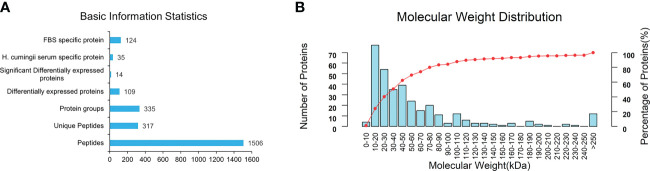
**(A)** Basic information and statistics of serum. **(B)** Coverage of protein by the identified peptides.

By searching against the *H.cumingii* and FBS Uniprot database, a total of 1506 peptides and 335 proteins were obtained (**Figure 5A**). A total of 109 DEPs were screened, including 14 significant DEPs (**Figure 5A**). Compared with FBS, there were 35 specific proteins in HCS. The distribution diagram of the relative molecular weight of proteins showed that 10-20 kDa proteins were the most abundant (**Figure 5B**).

### 3.5 DEPs and database enrichment analysis

**Figure 6:**
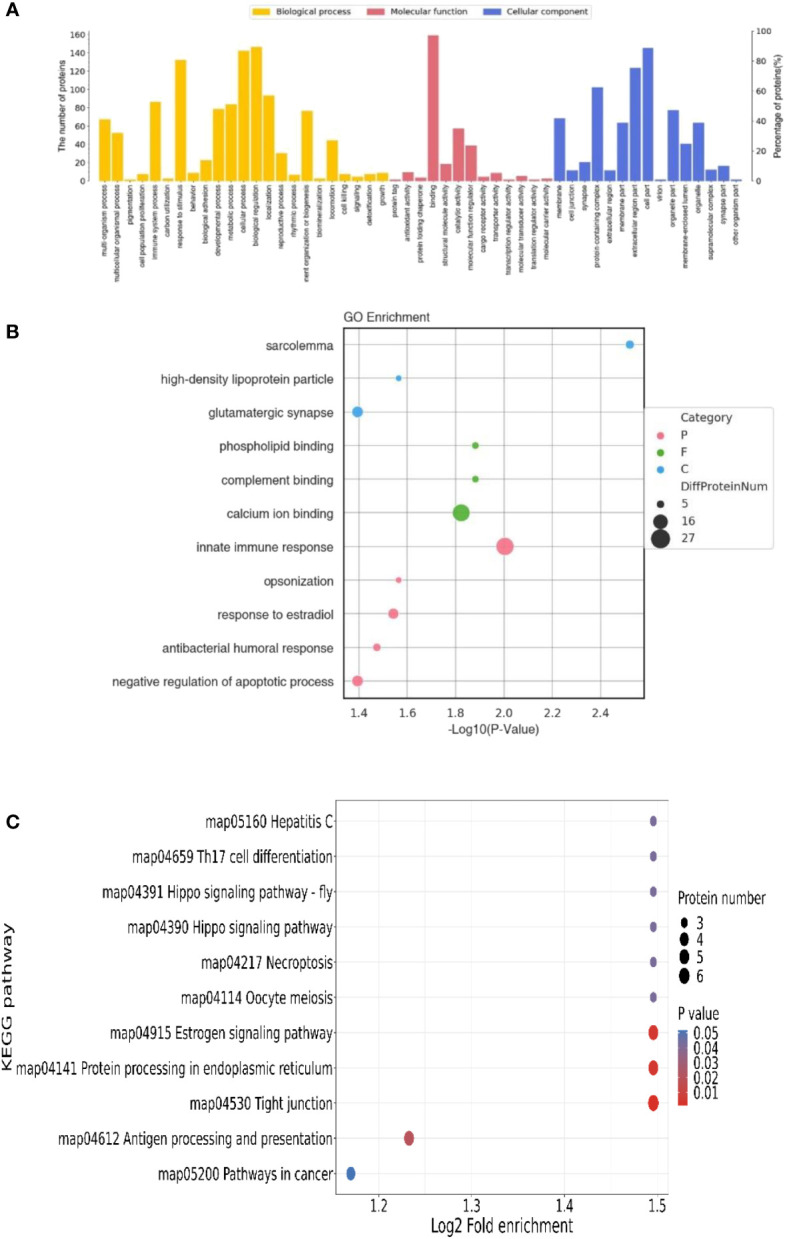
Basic statistics of DEPs in serum. **(A)** GO profiling of annotated DEPs. **(B)** GO enrichment of annotated DEPs. **(C)** KEGG enrichment of annotated DEPs.

The abundance of screened DEPs was evaluated by GO analysis. The proteins were classified by GO annotation based on three categories: molecular function (GO-MF), cellular component (GO-CC), and biological process (GO-BP, **Figure 6A**). DEPs were annotated by GO-BP for immune, growth, cellular and developmental processes, among which the number of proteins is related to biological regulation and cellular process. Response to the stimulus was significantly higher (*P*<0.05). GO-MF describes activation that occurs at the molecular level. The number of bindings-related DEPs was significantly higher than that of other groups (*P*<0.05). GO-CC annotation analysis showed that DEPs were distributed in different components of cells. The number of DEPs in the cell part and extracellular region part was significantly higher than in other groups (*P*<0.05). GO enrichment showed that innate immune response and calcium ion binding had the largest number of DEPs (**Figure 6B**, *P*<0.05).

The statistically significant DEPs identified in the KEGG pathway enrichment analysis are shown in **Figure 6C** (*P*<0.05). DEP distribution in immune inflammation, cell proliferation, differentiation, and metabolic absorption pathways were enriched. The enrichment in the immune and antigen presentation pathways was significantly higher than in other pathways (*P*<0.05).

As shown in **Supplementary Table 1**, in combination with GO and KEGG functional annotations, among the 109 DEPs and 64 *H. cumingii* unique proteins, 37 immune-related proteins were screened out, including HSPs (HSP90AB1, Hsc71, and HSPA8), lectin (CLEC11A, LGALS3BP, and MASP1), complement (C3, C8G, and C1R), Recombinant Transferrin (TF), Keratin (KRT1, KRT10, and KRT6A), etc. Of these, C1qDC6, α2M, TET1, IGL@, GST, and JCHAIN proteins were the unique protein of HCS. Most of the 13 DEPs associated with growth were growth-related proteins (IGF, EF-1α, MST1, TGFBI, and PPP2CA), among which YWHAE (14–3–3), PPP2CA, and LOC101902760 were involved in cell cycle-related pathway. Among the 9 DEPs related to biological mineralization, including ACTA1, RBP4, ACTB, CDH6, RGN, CaM, CGI_10018970, and sarcoplasmic calcium-binding protein, perlucin, mainly focus on the cytoskeleton, apolipoprotein, and calcium signaling pathways.

### 3.6 Protein validation by PRM

**Figure 7:**
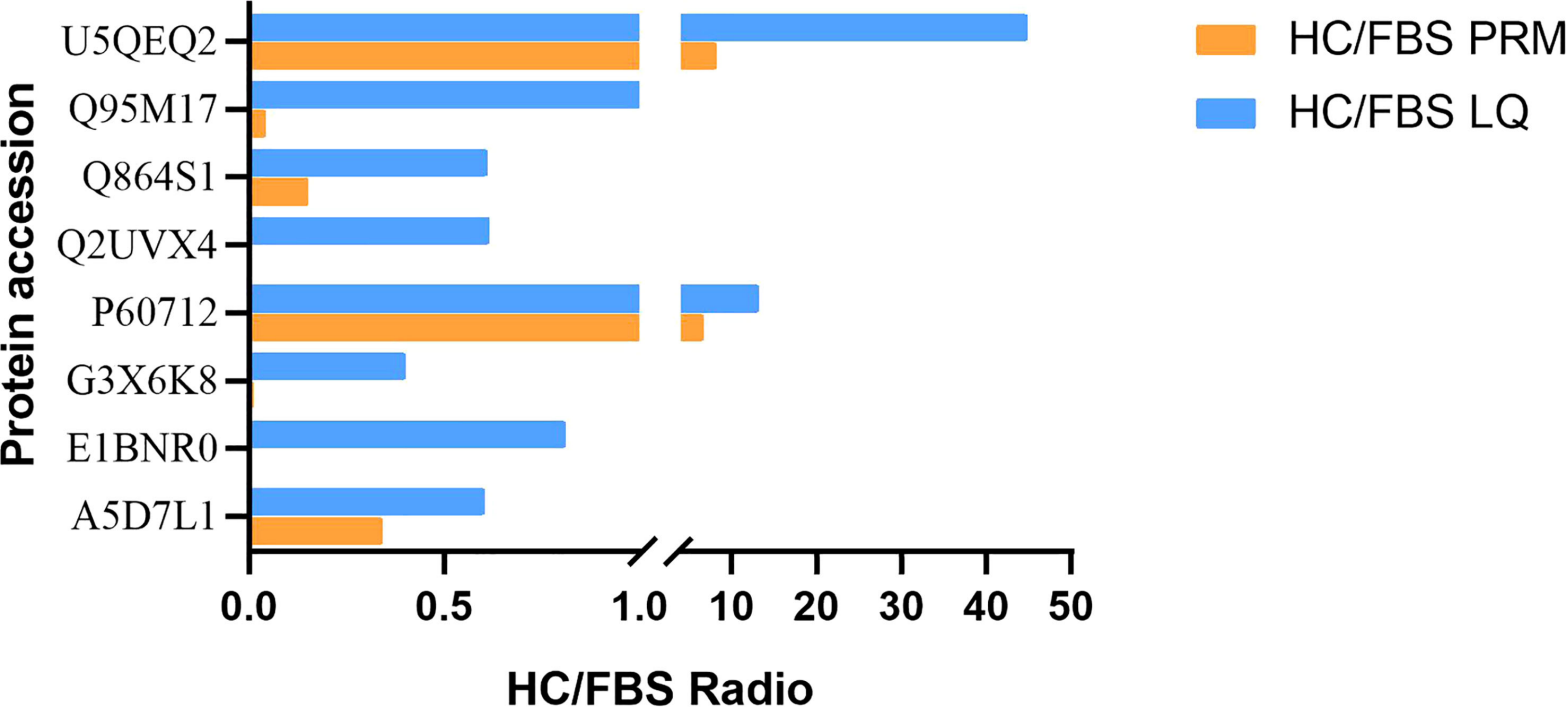
Confirmation of differentially expressed proteins detected in label-free analysis with PRM assays. HC/FBS radio indicates the relative abundance in the *H.cumingii* serum to that in the FBS. For full protein names, please refer to **Supplementary Table 1**.

By selecting proteins with significant differences between HCS and FBS, 20 DEPs were quantified to verify the label-free based proteomics data, including selected proteins related to immune metabolism and growth (**Supplementary Table 1**). PRM was used to quantify the selected proteins in 2 groups of serum samples (3 parallel groups), and 19 were quantified. The 11 proteins that were successfully verified were unique (**Table 1**), and 8 of them were immune response-related proteins, such as C1R, C8G, VCAM1, etc. Based on the multiple of significance difference, the difference of PRM between 8 selected significant proteins and label-free results was compared, and there was a significant difference between P60712 and U5QEQ2 (**Figure 7**, *P*<0.01)

**Table 1:** PRM validation of unique proteins.

Protein ID	Gene name	Function
A0A3Q1MG08	Mannose-binding lectin-related serine protease1(MASP1)	Immune response
A0A3Q1MGP1	C1R	Immune response
A0A3Q1MR54	C8G	Immune response
A7E3W2	Galectin-3-binding protein (LGALS3BP)	Immune response
E1BJ49	Recombinant Mannose Associated Serine Protease 2(MASP2)	Immune response
P08169	Insulin-like growth factor 1(IGF1)	Growth
P22226	Cathelicidin-1(CATHL1)	Immune response
Q0QES8	Glyceraldehyde-3-phosphate dehydrogenase (GAPDH)	Immune response
Q3SWW8	Thrombospondin-4 (THBS4)	Growth
Q5DPW9	Cystatin 6(CST6)	Growth
Q9GKR3	Vascular cell adhesion molecule 1(VCAM1)	Immune response

### 3.7 Analysis of DEPs related to immune responses

**Figure 8:**
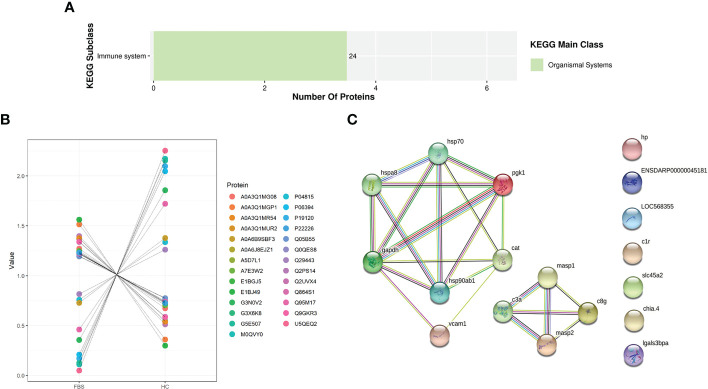
**(A)** KEGG Enrichment Bar Plot. **(B)** Differential proteins scatter plot. **(C)** Five strong interaction groups of the whole protein-protein interaction network analyzed by String software. Network nodes represent proteins. Edges represent protein-protein associations. Different colored lines represent the existence of the seven types of evidence used in predicting the associations. A red line indicates the presence of gene fusion evidence, a green line indicates gene neighborhood evidence, a blue line indicates gene co-occurrence evidence, a purple line indicates experimental evidence, a yellow line indicates text-mining evidence, a light blue line indicates database evidence, and a black line indicates co-expression evidence.

From label-free screening, we found that the number of proteins differentially expressed in immune response was the largest in *H.cumingii* and FBS. In this study, proteins related to immune response and identified DEPs related to innate and acquired immunity were mainly focused (**Supplementary Table 1**). The KEGG pathway enrichment Bar Plot showed that the number of DEPs was the most enriched protein in the immune system pathway (**Figure 8A**). After further screening, we marked scatter plot analysis. Ten up-regulated proteins and 17 down-regulated proteins were identified among the selected immune-related DEPs (**Figure 8B**). Proteins interact with other proteins in biological functions, and these interactions can be reflected in the biological process of GO. Therefore, proteins involved in the GO and KEGG enrichment pathways and immune-related pathways were selected (**Supplementary Table 1**). Thirty-three proteins were selected for PPI analysis, and 15 of these were included in the network (**Figure 8C**). There are two groups of related proteins, one of which has eleven, such as insulin-like growth factor 1 (IGF1), glyceraldehyde-3-phosphate dehydrogenase, and phosphoglycerate kinase 1. The other one has four, including mannan-binding lectin serine protease 1, mannan-binding lectin serine protease 2, complement component C3a, and complement component C8γ chain precursor.

## 4 Discussion

Because of the uncertainty of the medium, it is difficult to achieve a long-term culture of mussel cells *in vitro*. FBS is usually selected as a nutrient supplement in cell culture. Takeuchi cultured foot cells of Mediterranean mussel in L-15 medium supplemented with 20% FBS (34). FBS (2%), vitamin E (1.75 mg/L), and insulin (50 mg/L) could maintain the viability of mussel larval cells cultured *in vitro* for more than 2 months (9). Recently, a mixture of FBS and autologous serum has been used to improve cell survival time. The mantle cells were cultured in DMEM basal medium supplemented with 20% FBS and 10% HCS, and the cells could survive *in vitro* for more than 7 d (35). When cultivating tilapia macrophages, comparative observation of autologous serum and FBS training situation resulted in the containing 10% autologous serum growing well in culture medium, culture medium of FBS in cell death (36), showing that FBS was not suitable for tilapia macrophages *in vitro*, which is consistent with the results of this study.

Growth factors can promote cell growth (14) and immune factors are associated with cell survival. Pollution is a significant issue that needs to be addressed because mussel cells cannot develop *in vitro* for an extended period (20). Serum contains a large number of immune-related proteins, and immune factors play an important role in pollution prevention and control. For vertebrates, only innate immunity protects against invading pathogenic microorganisms (37). As a lower invertebrate, *H. cumingii* can complete non-specific immune responses under the action of immune factors such as lectins, complement, and HSPs (21). Humoral immunity plays an important role in innate immunity (12), and serum is an important component of humoral immunity. Differential proteomic analysis between the two groups revealed that several immune and growth pathway-related genes (lectin, HSP, and calreticulin) were annotated. These genes were significantly correlated with MAPK, PI3K-AKT, NF-κB, and calcium signaling pathways. It was further verified that antigen processing and presentation (38), estrogen signaling pathway (39), and other pathways were enriched in this study. It plays a role in regulation and intracellular trafficking, thus contributing to immunogenicity and cellular tolerance. Therefore, serum participates in cell proliferation and plays an important role in various physiological and pathological processes, such as cell phagocytosis, cell apoptosis, and inflammatory response in cell culture.

HSPs are endogenous protective proteins. This family of proteins plays an important role in protecting cells from stress damage (40), participating in immune response and autoimmunity (41). High expression of heat shock protein 70 (HSP70) can protect human monocytes from toxicity (42) and apoptosis (43) caused by hydrogen peroxide (H_2_O_2_) and protect human A-431 cells from sodium cyanide damage (44). There is a large amount of HSP70 protein distribution in the gill cells of *Pomacea canaliculata*, which is closely related to the physiological function of gill tissue cells (13). Our study showed that HSP70 was highly expressed in the homemade serum of *H. cumingii*, and its content was significantly higher than that of FBS, as verified by PRM. After the cells were invaded by bacteria, HSP70 in the homemade serum was involved in the immune response in cell culture, protecting cells and making cells having certain self-defense abilities, and causing anti-infection immunity, which is crucial for the survival of cells. HSP70 can interact with a variety of apoptotic molecules. Up-regulation of HSP70 expression can inhibit p53-induced apoptosis (45), which explains that the cell culture survival time of the self-made serum in this study is better than that of the FBS group, and HSP70 may play an important role in cell proliferation by regulating or participating in DNA synthesis. HSPA protects the body by promoting protein synthesis and changing its spatial structure when encountering external adverse factors and environmental changes, and participates in the regulation of immune system function, cell differentiation and transport (46). The expression of HSPA8 in autologous serum was higher than that in FBS, which suggested that autologous serum is better than FBS when it is added to invertebrate cell culture system.

The complement system exists widely in invertebrates (47) and plays an important role in immune response, among which the lectin pathway is considered the most primitive complement pathway (48). Lectins play an important role in apoptosis and innate immunity (49). Key components of the lectin pathway, including C3 and mannan-binding lectins serine peptidase (MASP), have been identified in oysters *Crassostrea Gigas*. Blood cells from oysters injected with the C-type lectin gene (*CgCLec-2*) significantly increased the phagocytosis of pathogens (50), which confirms that lectins play an important role in pathogen clearance and complement system activation. C-type lectin gene (C3) has been identified as a processed protein in acellular hemolymph and plays a key role in antimicrobial immunity in mollusks (51). MASP1 and MASP2 are the core proteases in the complement system and are required for lectin pathway activation (46). MASP promotes C3 cleavage by interacting with complement component C3. C3 b-mediated phagocytosis is the most important function of the invertebrate complement system (51). The C1qDC of *H. cumingii* can participate in the anti-bacterial immune response (12). In this study, there were significant differences in complement components. C1qDC is a unique protein in the autologous serum. C3 is activated in shellfish cell culture to induce mantle cells to phagocytose opsonized pathogens. C1qDC is involved in innate immune response, thus making the cells have a stronger immune tolerance mechanism.

Calreticulin (CALR) is mainly localized in the endoplasmic reticulum (ER) and is involved in ER glycoprotein folding and calcium homeostasis, as well as cell proliferation, phagocytosis, and apoptosis (52). Knockdown of CALR expression in HSC-LX2 cells can promote cell apoptosis, which explains the reason for selecting autologous serum in this study (53). The formation of pearls is related to calcium ions. As an important part of the formation of pearls, the mantle is involved in the transport, enrichment, and secretion of Ca^2+^ (54). The proteins annotated are concentrated in the calcium ion binding pathway in this study. Calcium is required in mantle cell culture. Apolipoprotein is the main component of plasma lipoprotein. APOB recognizes low-density lipoprotein receptor (LDLR) and is the main protein constituting it (46). The cellular catabolic lipoprotein and lipoprotein receptor binding on the cell membrane are affected by apolipoproteins (55). APOB, as an upregulated protein, was significantly differentially expressed in the two serums, mainly involved in biological processes, such as the regulation of lipoprotein oxidation and immune system response (55) and may be involved in the regulation and management of lipid metabolism in mussel cell culture.

YWHAE (14-3-3E) acts as molecular chaperones (56), which facilitates the movement of phosphorylated heat shock factor 1 from the nucleus to the cytoplasm, increasing cell heat adaptation (33). It also links signal transduction and cell proliferation, promoting cell growth (57). In this study, the expression of the upregulation protein in the HCS was significantly higher than that in FBS, which indicates that the upregulation protein in the HCS plays an important biological role.

There were 26 differentially upregulated proteins in the HCS. Most of the up-regulated proteins were enriched in the aspect of the immune response, which could improve the antioxidant capacity in the process of cell culture, make the cells have strong immune tolerance, and increase the survival time. It is found that the effect of autologous serum in the cell culture of *H. cumingii* is better than that of FBS. Therefore, the autologous serum of *H. cumingii* contains a variety of immune factors such as HSPs, C3, CALR and so on, which can be added as one of the components of culture medium in freshwater mussel cell culture. Thus, further studies are needed to determine whether adding additional immune factors in mussel cell culture can improve cellular immune resistance.

## 5 Conclusion

Label-free quantitative proteomic method analysis was used to analyze and compare the differences in protein components between autologous serum of *H. cumingii* and FBS. It was found that the differentially expressed proteins were mainly involved in immune regulation and cell growth, and there were *H. cumingii* specific proteins involved in various biological pathways such as metabolism, cell differentiation and mineralization, thus promoting the survival and growth of mussel cells. It revealed the reason that autologous serum was superior to FBS system in mussel cell culture medium. This study analyzed the important role of immune factors in cell culture, which made autologous serum a necessary component of mussel cell culture, which provided a fresh viewpoint on the selection of FBS and autologous serum, and also provided a new theoretical basis for further establishing mussel cell lines.

## Data availability statement

The mass spectrometry proteomics data have been deposited to the ProteomeXchange Consortium (http://proteomecentral.proteomexchange.org) *via* the iProX partner repository with the dataset identifier PXD041482.

## Ethics statement

The animal study was reviewed and approved by Shanghai Ocean university.

## Author contributions

ZB and HW: carried out the experiments, data analysis and paper writing. XL: investigation and formal analysis. XS: bioinformatics analysis. YC: data recording. YF: paper writing and revision. WL: meticulously analyzed and experimental design. All authors contributed to the article and approved the submitted version.
