# Supplementary material for: Presence of immune factors in freshwater mussel (Hyriopsis cumingii) entails autologous serum an essential component in the culture of mantle cells
Source: Front Immunol. 2023 May 5;14:1173184. doi: 10.3389/fimmu.2023.1173184 (PMC10196017; doi:10.3389/fimmu.2023.1173184)
Supplement: Supplementary file 1 [file Table_1.docx]

Supplementary Table 1 DEPs between *H.cumingii* serum and FBS

| Protein ID | Gene name | HCS/FBS serum | | Up/Down | | Note |
| --- | --- | --- | --- | --- | --- | --- |
| **Immune response** |  |  |  | |  | |
| Q29443 | Recombinant Transferrin (TF) | 1.544 | Up | |  | |
| A0A140T897 | Albumin (ALB) | 19.315 | Up | |  | |
| G5E507 | HSP90AB1 | 17.023 | Up | |  | |
| E1BNR0 | Apolipoprotein (APOB) | 10.063 | Up | | PRM | |
| Q2UVX4 | C3 | 0.612 | Down | | PRM | |
| A0A3Q1MG08 | Recombinant Mannose Associated Serine Protease1(MASP1) | 0.53 | Down | | PRM | |
| E1BJ49 | Recombinant Mannose Associated Serine Protease 2(MASP2) | 0.192 | Down | | PRM | |
| G3X6K8 | HP | 0.399 | Down | | PRM | |
| U5QEQ2 | Heat shock cognate70(Hsc70) | 44.714 | Up | | PRM | |
| A5D7L1 | Recombinant C-Type Lectin Domain Family 11, Member A (CLEC11A) | 0.6 | Down | | PRM | |
| Q95M17 | CHIA | 3.731 | Up | | PRM | |
| P04815 | Spleen trypsin inhibitor I | 1.76 | Up | |  | |
| A0A3Q1MUR2 | Ig-like domain-containing protein | 0.579 | Down | |  | |
| Q864S1 | Cathepsin C (Fragment) | 0.608 | Down | | PRM | |
| A7E3W2 | Galectin-3-binding protein (LGALS3BP) | 0.564 | Down | | PRM | |
| A0A6J8EJZ1 | MCOR_53061 | 0.596 | Down | |  | |
| P22226 | Cathelicidin-1(CATHL1) | 0.59 | Down | | PRM | |
| Q0QES8 | Glyceraldehyde-3-phosphate dehydrogenase (GAPDH) | 0.439 | Down | | PRM | |
| Q9GKR3 | Vascular cell adhesion molecule 1(VCAM1) | 0.436 | Down | | PRM | |
| A0A3Q1MR54 | C8G | 0.393 | Down | | PRM | |
| A0A3Q1MGP1 | C1R | 0.238 | Down | | PRM | |
| P19120 | Heat shock cognate 71 kDa (HSPA8) | 12.276 | Up | |  | |
| A0A6B9SBF3 | Ig heavy chain variable region (Fragment) | 1.894 | Up | |  | |
| Q05B55 | Immunoglobulin kappa (IGK) | 0.648 | Down | |  | |
| E1BGJ5 | CD93 | 0.582 | Down | |  | |
| G3N0V2 | Keratin 1 (KRT1) | 5.192 | Up | |  | |
| P06394 | Keratin 10(KRT10) | 9.747 | Up | |  | |
| M0QVY0 | Keratin 6A(KRT6A) | 19.281 | Up | |  | |
| Q3MHM5 | TUBB4B | 0.016 | Down | |  | |
| A0A6J8DHD9 | MCOR_40368 |  |  | | *H.C* Unique | |
| A0A223HH94 | C1q-domain-containing(C1qDC6) |  |  | | *H.C* Unique | |
| A0A1G5 | α2-Macroglobulin (α2M) |  |  | | *H.C* Unique | |
| F1MGK2 | Ten-eleven translocation1(TET1) |  |  | | *H.C* Unique | |
| Q3T101 | Immunoglobulin lambda locus (IGL@) |  |  | | *H.C* Unique | |
| A8UMW1 | Glutathione S-transferases (GST) |  |  | | *H.C* Unique | |
| Q3SYR8 | JCHAIN |  |  | | *H.C* Unique | |
| J9U877 | Calreticulin (CALR) |  |  | | *H.C* Unique | |
| **Growth** |  |  |  | |  | |
| A0A3Q1M119 | 14-3-3 epsilon isoform (YWHAE) | 9.63 | Up | |  | |
| P08169 | Insulin-like growth factor 1 (IGF1) | 0.42 | Down | | PRM | |
| Q3SWW8 | Thrombospondin-4 (THBS4) | 0.263 | Down | | PRM | |
| D9IWS1 | Elongation factor (EF-1 α) | 20.291 | Up | |  | |
| P13384 | Insulin-like growth factor-binding protein-1(IGFBP1) | 0.532 | Down | |  | |
| A0A3Q1LSX3 | Mammalian Sterile20-like Kinase (MST1) | 1.617 | Up | |  | |
| A0A3Q1MNL9 | Transforming growth factor beta induced (TGFBI) | 0.25 | Down | |  | |
| Q5DPW9 | Cystatin 6(CST6) | 0.454 | Down | | PRM | |
| A0A3Q1M4K3 | LOC101902760 |  |  | | *H.C* Unique | |
| A0A6C0W967 | Cyclophilin A (CypA) |  |  | | *H.C* Unique | |
| A0A6J8CE23 | MCOR_28694 |  |  | | *H.C* Unique | |
| Q58D70 | Protein phosphatase 2 catalytic subunit alpha (PPP2CA) |  |  | | *H.C* Unique | |
| Q704W6 | Signal transducer and activator of transcription(stat3) |  |  | | *H.C* Unique | |
| **Biomineralization** |  |  |  | |  | |
| A4IFM8 | Alpha-actin (ACTA1) | 0.025 | Down | |  | |
| P18902 | Retinol binding protein 4(RBP4) | 7.818 | Up | |  | |
| P60712 | β-Actin (ACTB) | 13.06 | Up | | PRM | |
| A0A3Q1LTJ1 | Cadherin-6(CDH6) |  |  | | *H.C* Unique | |
| Q9TTJ5 | Regucalcin (RGN) |  |  | | *H.C* Unique | |
| A0A3Q1MR28 | Calmodulin (CaM) |  |  | | *H.C* Unique | |
| A0A6J8BH95 | CGI_10018970 |  |  | | *H.C* Unique | |
| O61284 | Sarcoplasmic calcium-binding protein |  |  | | *H.C* Unique | |
| A0A161VF18 | Perlucin |  |  | | *H.C* Unique | |
